# A novel mutation in the glycine decarboxylase gene in patient with non-ketotic hyperglycinemia

**DOI:** 10.17712/nsj.2017.2.20160468

**Published:** 2017-04

**Authors:** Engin Kose, Uluc Yis, Semra Hiz, Nur Arslan

**Affiliations:** *From the Division of Pediatric Metabolism and Nutrition (Arslan, Kose), Division of Pediatric Neurology (Yis, Hiz), Department of Pediatrics, Faculty of Medicine, Dokuz Eylul University, and from Izmir Biomedicine and Genome Center (Arslan), Izmir, Turkey*

## Abstract

Non-ketotic hyperglycinemia (NKH) is a rare inborn error of metabolism and is caused by a glycine cleavage system deficiency. Eighty-five percent of patients present with the neonatal type of NKH, the infants initially develop lethargy, seizures, and episodes of apnea, and most often death. Between 60-90% of cases are caused by mutations in the glycine decarboxylase (GLDC). We believed that more mutation reports especially for rare disease as NKH help to evaluate the genotype-phenotype relationship in patients with GLDC. In this study, we describe a case of a neonate admitted to intensive care unit with hypotonia, respiratory failure, lethargy, poor feeding. Due to the history of 2 non-ketotic hyperglycinemia diagnosed male siblings, molecular prenatal diagnosis in patient was performed and a novel c.2963G>A (Arg998Gln) homozygous mutation within the GLDC gene has been detected. We aimed to contribute to mutation knowledge pool of GLDC gene with a novel mutation.

Non-ketotic hyperglycinemia (NKH) (OMIM #605899) is an autosomal recessive inborn error of metabolism and is caused by a glycine cleavage system deficiency, resulting in high levels of glycine in all tissues including the brain.^1^,^2^ In the neonatal type of NKH, the infants initially develop lethargy, seizures, and usually rapidly progress to myoclonic seizures, episodes of apnea, and most often death. Fifteen Percent of patients present with late onset milder form.^3^ In many patients, clinical features, electroencephalography (burst-suppression-like pattern) and brain magnetic resonance imaging (MRI) findings (hypoplasia of corpus callosum) gives an idea for NKH. In addition to these findings, genetic investigation has an importance in the diagnosis of disease. It can also help in providing prenatal diagnosis in affected families. Glycine decarboxylase (GLDC) (accounting for the 60-90% of disease), aminomethyltransferase (AMT) (accounting the 20% of disease), and hydrogen carrier protein (GCSH) (accounting for <1% of disease) are three genes in which biallelic mutations are known to cause NKH.^1^,^4^ Here we describe a case of a neonate with NKH in which a novel c.2963G>A (Arg998Gln) homozygous mutation within the GLDC gene has been detected.

Case Report. A 3-month-old Turkish girl was admitted to our Intensive Care Unit at the age of 2 days with hypotonia and respiratory distress. The baby weighting 3000 gr was born to third-degree consanguineous parents after a full-term uneventful pregnancy. Her parents noted history of deaths of 2 non-ketotic hyperglycinemia diagnosed male offspring on 5 months and 11 months of age. Due to history of NKH diagnosed siblings, before admitting to our center, molecular prenatal diagnosis in patient and genetic investigation in her parents were performed and c.2963G>A (Arg998Gln) homozygous mutation and c.2963G>A (Arg998Gln) heterozygous mutation were identified in the GLDC gene (in exon 25) (**Figure 1**). This is a novel mutation and because it affects a conserved amino acid, it was considered pathogenic. Termination of pregnancy was recommended by genetic counseling, but parents refused.

**Figure 1:**
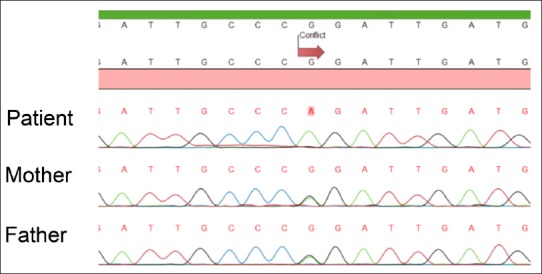
- Sequence analysis of the patient’s DNA shows a homozygous mutation. The part of sequence analysis is from part of exon 25, showing G>A change at 2963, resulting in Arg998Gln amino acid replacement. The parents are heterozygous for the same change.

On admission, she had progressive hypotonia, lethargy, poor feeding and respiratory distress. No dysmorphic appearance was noted and other system examinations were unremarkable. Laboratory investigations showed respiratory acidosis (pH: 7.18, pCO_2_: 69.2 mmHg, HCO_3_: 19.6 mmol/L, base excess: -2.5 mmol/L). Serum sodium, urea, creatinine levels and other biochemical parameters were normal. Serum C-reactive protein was negative. Blood glucose, serum lactate, ammonia and complete blood count were unremarkable. In urinary analysis ketone was negative. Plasma and urine amino acid analysis, urine organic acid analysis were also performed. Compatible with NKH, glycine levels were found elevated in plasma and urine [2048 µmol/L (20-400) and 35160 µmol/g creatine (0-7500)]. Organic acidemias were ruled out with normal urine organic acid analysis, blood ammonia level, and negative urine ketone. Urine and blood cultures were negative. Lumbar puncture was not performed. Electroencephalography (at 22th day of life) indicated a normal background activity with sparse spikes in the right temporoparietooccipitial region. Based on clinical and laboratory findings, molecular analysis and familial history, a diagnosis of NKHG was made. Patient was intubated and supported with mechanical ventilation in the first day of admission to intensive care unit. Sodium benzoate (500 mg/kg/day), phenobarbital (5 mg/kg/day), folinic acid (15 mg/day) treatments were initiated. Protein intake reduced to 1 gr/kg/day and adequate caloric intake was provided to prevent catabolism. In the 12th day of follow-up, she was extubated. With the sodium benzoate, phenobarbital and protein restricted diet treatments blood glycine level reduced to normal range [280 µmol/L (20-400)]. At age 3 months, cranial magnetic resonance imaging was performed and hypoplasia of corpus callosum was revealed. At the present follow-up (10 months) she is seizure free with phenobarbital, sodium benzoate, folinic acid and protein restricted diet medications.

## Mutation analysis

The GLDC gene mutation analysis was performed by sequencing of the coding exons of the gene. Genomic DNA was isolated from peripheral blood and amniotic fluid cells by standard techniques. PCR was performed to amplify the exons of GLDC gene. All PCR products were sequenced by the dye termination method using a DNA sequencing kit (Perkin-Elmer, Foster California, USA) and analyzed using The ABI Prism 3100 sequence analyzer (Applied Biosystems, Foster, California, USA).

## Discussion

We reported a patient with a diagnosis of NKH, due to a novel c.2963G>A (Arg998Gln) homozygous mutation state in exon 25 in GLDC gene. In our patient, NKH diagnosed siblings, consanguineous parents, clinical and laboratory findings strongly supported the pathogenicity of the mutation.

Non-ketotic hyperglycinemia manifests as an acute neonatal encephalopathy in the majority of patients, and 15% of patients present with late onset milder form. In the neonatal type of NKH, the infants initially develop lethargy, seizures, and usually rapidly progress to myoclonic seizures, episodes of apnea, and most often death.^1^,^2^ In our patient, consistent with the literature, lethargy, poor feeding, muscle weakness, breathing difficulty occurred in the second day of life. In laboratory investigation, elevated glycine level in blood and urine were compatible with NKH. Furthermore, her siblings died due to NKH in 5 months and 11 months of age. With these findings we unhesitatingly diagnosed the neonatal severe form of NKH in our patient.

Non-ketotic hyperglycinemia is an inherited metabolic disease that is due to a defect in glycine cleavage enzyme that arises due to absent or decreased activity of the glycine cleavage system. Enzyme consists of four protein component named as P- (glycine decarboxylase), T- (aminomethyltransferase), L- and H- (hydrogen carrier protein) proteins. There are 60-90% of cases caused by mutations in the P protein also known as GLDC.^5^ Approximately 20% of GLDC mutant alleles are exonic/multiexonic deletions or duplications. In Finnish patients, Ser564Ile and Gly762Arg mutations in GLDC gene have been identified in 70% of alleles and 8% of alleles. Many other mutations have been described in single individuals.^1^,^5^,^7^,^8^ In our patient, molecular prenatal diagnosis was performed and an association between the neonatal severe form of NKH patients were presented in literature and Arg998Gln homozygous mutation in the GLDC gene was described. To the best of our knowledge, Arg998Gln mutation in GLDC gene is firstly described in this patient. Although, there is no evidence of correlation between genotype and phenotype in NKH,^9^ according to clinical manifestations of our patient and her siblings, we can speculate that that Arg998Gln mutation in GLCD gene causes the severe neonatal type of NKH. In NKH, neurological manifestations are caused by N-methyl-D-aspartate receptor overstimulation. Sodium benzoate, antiepileptic medications and dextromethorphan are currently used in the treatment of epilepsy due to NKH.^10^ In our patient, treatment with sodium benzoate and phenobarbital was effective against epileptic activity. At the last follow-up, she is seizure-free without any specific medication for 10 months.

In conclusion, in this report, we describe a novel association between Arg998Gln homozygous mutation in the GLDC gene in a Turkish patient with the neonatal type of nonketotic hyperglycinemia. We believed that more mutation reports, analysis of enzyme activity and functional effect of mutations help to evaluate the genotype-phenotype relationship in patients with GLDC. Further studies are needed to determine the impact of Arg998Gln mutation on protein structure and function.
